# Time to rethink routine histopathology after adult circumcision? Long-term oncological outcomes from an 11-year cohort

**DOI:** 10.1007/s00345-026-06432-y

**Published:** 2026-04-30

**Authors:** Ghulam Mustafa Nandwani, Mubeshar Hassan, Devanand Lohana, Prasad Bollina

**Affiliations:** Department of Urology, Ninewells Hospital and Medical School, Dundee, Scotland, UK

**Keywords:** Circumcision, Prepuce, Foreskin, Penile intraepithelial neoplasia, Cost-benefit analysis

## Abstract

**Purpose:**

To evaluate whether routine histopathological examination of the prepuce following adult circumcision without clinical suspicion of malignancy influences patient management and healthcare resource utilisation, and to report circumcision frequency and long-term oncological outcomes.

**Methods:**

A retrospective cohort study was conducted of all adult circumcisions performed between January 2015 and December 2025. Patients aged ≥ 15 years undergoing circumcision for symptomatic phimosis, paraphimosis, or recurrent balanitis were included. Cases with suspected malignancy or known premalignant lesions were excluded. Demographic, operative, histopathological, and follow-up data were extracted from electronic records. Median follow-up was 85 months. Cost analysis was based on an estimated histopathology cost of £226 per specimen.

**Results:**

Of 1,648 listed patients, 1,523 met the inclusion criteria (median age 44 years). Phimosis was the primary indication (98.2%). Lichen sclerosus was identified in 62% of specimens, while incidental penile intraepithelial neoplasia (PeIN) was detected in 0.5%. No invasive carcinomas were identified. All PeIN cases were managed with circumcision alone, and no progression or additional intervention was required at a median follow-up of 81 months. Eliminating routine histopathology would have saved 304.6 consultant hours and £344,198 over 11 years (£31,291 annually).

**Conclusions:**

In the absence of clinical suspicion, routine histopathological examination of the prepuce rarely detects clinically significant malignancy or alters management. A selective, risk-adapted approach may optimise resource utilisation while maintaining oncological safety.

## Introduction

Circumcision is the commonly performed procedure for religious, cultural and medical reasons. Men > 15 years are mostly circumcised due to medical reasons. The global prevalence in > 15 years is 36.7%, with a very high prevalence where circumcision is performed for religious and cultural reasons [1, 2].

Routinely, prepuce histopathological examination is performed to assess pathological findings and, in particular, to rule out malignancy. Routine histopathological exams without suspicion of penile cancer were reportedly not beneficial [3]. Several studies have reported overwhelmingly benign findings, with most demonstrating nonspecific inflammation or LS [3–5]. The detection of PeIN or invasive carcinoma in the absence of suspicious clinical features appears to be uncommon. Furthermore, where such cases were identified, they were typically associated with visible lesions, induration, or ulceration that would have prompted histopathological assessment [6–9].

The practice of sending prepuce for histopathology after circumcision is variable, particularly where the procedure is done as part of the public service. This lack of uniformity reflects the absence of clear, evidence-based guidelines [1, 3]. The available literature is limited, often confined to single-centre series with relatively small patient cohorts and limited follow-up, restricting generalisability [1, 3, 10]. Consequently, uncertainty persists regarding whether routine histopathology meaningfully influences patient management when malignancy is not suspected. Each specimen submitted for histopathology requires both time and financial resources. In resource-limited settings, the impact is even more pronounced, where laboratory capacity may be better utilised with greater diagnostic or therapeutic importance [11, 12].

Literature offers limited evidence regarding the long-term benefits of routine histopathological examination of the prepuce. Consequently, surgeons remain hesitant to alter their current practice of routine histopathological requests following circumcision. Larger-scale studies are necessary to provide more robust evidence [17, 18, 19].

The aim of this study was to evaluate whether routine histopathological examination of the prepuce affected clinical management decisions and healthcare resource utilisation. Additionally, this research sought to provide the annual adult circumcisions and adverse oncological outcomes with follow-up.

## Methods

Data was extracted for all routine circumcisions from EPR for a cohort of patients between 1st January 2015 and 31st December 2025. Inclusion criteria were pathological symptomatic phimosis, paraphimosis and recurrent balanitis. Exclusion criteria were age < 15 years, known premalignant foreskin lesions, suspected penile cancer and where histopathology was not available.

Caldicott’s approval was obtained (approval number IGTCAL-2025-112); patient demographics, indications for procedure, operative findings, histopathological results, and follow-up information were collected. Follow up was conducted by reviewing the latest urology-related reviews and referrals on EPR. For study purposes, the average cost of histopathology was calculated at £226 by averaging the available costs from UK hospitals. Statistical analyses were performed using IBM SPSS Statistics Software version 22. Categorical variables were analysed using Fisher’s Exact Test or Chi-square tests, and continuous variables were summarised with medians and interquartile ranges.

## Results

A total of 1648 patients were listed for circumcision, and 125 (7.6%) were excluded. Malignancy was suspected in 4 (0.24%) at the time of booking for procedure while in 21 (1.3%) per-operative examination was suspicious. For detailed analysis of suspected cancer cases is presented in Table 1. Dorsal slit performed in 3 (0.2%), procedure not required in 8 (0.5%) as symptoms resolved, 3 (0.2%) did not attend for the procedure while in 86 (5.2%) histopathology was not requested.

**Table 1:** Histopathological details of patients with suspicion of malignancy at the time of circumcision n 25

All ages	Invasive SCC	Lichen Sclerosis	Inflammation	Others
n (%)	n (%)	n (%)	n (%)	n (%)
Age years – median (min – max), IQR; 56 (40–89), IQR 23				
	7 (28)	7 (28)	6 (24)	5 (20)
Age groups				
21–40 years, n 1 (4)	0	1 (4)	0	0
41–60 years, n 12 (48)	2 (8)	4 (16)	2 (8)	4 (16)
> 60 years, n 12 (48)	5 (20)	2 (8)	4 (16)	1 (4)
Age range in Invasive SCC 7 (28), median 72 (52–89), IQR 15.5				
Cancer suspicion n (%)				
Pre-procedure n 4 (16)	3 (12)	0	0	1 (4)
Per-op high n 11 (44)	3 (12)	3 (12)	2 (8)	3 (12)
Per-op low n 10 (40)	1 (4)	4 (16)	4 (16)	1 (4)

The final study population consisted of 1,523 patients, with a median age of 44 years (15–92), IQR 36 and a median follow-up of 85 months (range 0.6–132; IQR 73 months). Most patients (60%) were between 21 and 60 years old (Table 2). The primary indication for circumcision was phimosis (98%0.2). Histopathological analysis revealed LS in 62%, while PeIN was identified in 0.5% (Table 3).

**Table 2:** Indication of circumcision (n 1523)

Age groups, *n* (%)	Phimosis	Paraphimosis	Others
	*n* (%)	*n* (%)	*n* (%)
15–20 years, n 167 (11)	163 (97.6)	2 (1.2)	2 (1.2)
21–40 years, n 528 (34.7)	520 (98.5)	5 (0.9)	3 (0.6)
41–60 years, n 390 (25.6)	385 (98.7)	2 (0.5)	3 (0.8)
> 60 years, n 438 (28.8)	428 (97.7)	5 (1.1)	5 (1.1)
All ages, n 1523 (100)	1496 (92.2)	14 (0.9)	13 (0.9)

**Table 3:** Histopathological details of patients with no suspicion of malignancy at the time of circumcision

Age years – median (min – max), IQR			43 (15–92), 46			
Age groups	PeIN	LS	C Inflam	Others	Normal	Fibrosis
n (%)	n (%)	n (%)	n (%)	n (%)	n (%)	n (%)
15–20 years	1 (0.6)	65 (38.9)	88 (52.7)	1 (0.6)	9 (5.4)	3 (1.8)
167 (11)						
21–40 years	3 (0.6)	292 (55.3)	208 (39.4)	6 (1.1)	12 (2.3)	7 (1.3)
528 (34.7)						
41–60 years	1 (0.3)	281 (72.1)	103 (26.4)	1 (0.3)	1 (0.3)	3 (0.8)
390 (25.6)						
> 60 years	2 (0.5)	302 (68.9)	131 (29.9)	3 (0.7)	0	0
438 (28.8)						
All ages	7 (0.5)	940 (61.7)	530 (34.8)	11(0.7)	22 (1.4)	13 (0.9)
1523 (100)						

*PeIN* Penile Intraepithelial Neoplasia, *LS* Lichen Sclerosis, *C Inflam* Chronic Inflammation and *Normal* No significant abnormality in prepuce

In PeIN, 4 (58.2%) and 3 (42.9%) were smokers and diabetics, respectively while 2 (28.6) had Psoriasis. PeIN was found in 58.2% under 40 years and all cases were reviewed in a multidisciplinary team meeting. Patients were advised to perform regular self-examinations and were scheduled for clinical review if any new changes or concerns were identified during self-examination. Since there were no lesions involving the penile shaft or glans, circumcision alone was considered sufficient for complete removal of incidental PeIN. At a median follow-up of 81 months (range 28–128; IQR 55 months), no patients required additional assessment based on self-examination findings. Detailed age distribution and histopathological characteristics of PeIN cases are presented in Table 4.

**Table 4:** Histopathological details of patients with PeIN

Age years – median (min – max), IQR			38 (20–78), 30.5		
Age	Grade	Inflammation	Lichen Sclerosis	HPV	Other
Years	n (%)	n (%)	n (%)	n (%)	n (%)
27	Differentiated	-	-	-	-
55	Differentiated	-	Yes	-	-
38	Undifferentiated	Zoon’s	-	Yes	Psoriasis
20	Differentiated	-	-	-	-
78	Differentiated	Yes	-	-	-
62	Undifferentiated	-	-	-	-
29	Undifferentiated	-	Yes	Yes	-

## Discussion

The routine histopathological examination of the prepuce following adult circumcision remains a subject of ongoing debate [3, 10, 13]. This study, comprising a large cohort of 1523 patients with extended follow-up, provides valuable insight into the prevalence of significant pathological findings, specifically premalignant lesions in the foreskin where neither preoperative nor intraoperative suspicion of malignancy existed. By critically assessing the necessity and value of routine histopathological analysis, our findings contribute to the evolving discourse on optimising patient care, resource utilisation, and cost-effectiveness in urological surgery.

PeIN and LS are recognised entities with potential for invasive malignant transformation, albeit with varying degrees of risk [6]. Established risk factors for PeIN include the presence of prepuce, phimosis, suboptimal genital hygiene, tobacco use, chronic inflammation, multiple sexual partners, and HPV infection. Most premalignant lesions are located on the glans penis, and up to 30% may progress to invasive carcinoma if left untreated. However, moderate dysplasia tends to follow a benign course, with malignant transformation occurring in approximately 1% of cases [7, 8]. LS, while considered a premalignant condition, lacks consensus regarding optimal surveillance strategies, though regular clinical examination is widely recommended [14]. In line with contemporary practice, all patients in this study received counselling on self-examination and were referred for further assessment only if new or suspicious changes were noted.

The principal question addressed by this study is whether routine prepuce histopathological evaluation following routine circumcision yields clinically meaningful findings that alter patient management. Among our cohort, incidental PeIN was identified in only 0.5% of cases, and no cases of invasive carcinoma were detected. LS was the most prevalent diagnosis, identified in 62% of specimens, while inflammation represented the next most common finding. Importantly, all patients with incidental PeIN were managed successfully with circumcision alone, and no subsequent invasive cancers were observed during a median follow-up of 81 months. These findings suggest that, in the absence of clinical concern, routine histopathological examination rarely identifies additional pathological findings that would change the therapeutic approach.

A review of the literature supports these observations. Khalabazyane et al. evaluated 334 circumcisions and found that, among 325 patients without clinical suspicion of malignancy, no cases of cancer were identified histologically; LS was the most frequent diagnosis (60.6%) [10]. Shah et al. reported on 147 patients, with histopathology performed in 69%; inflammation predominated, and only one case of PeIN was detected with HIV and phimosis, but without clinical suspicion of cancer in normal foreskin [13]. Similarly, Kerr et al. analysed 508 cases, with histopathology ordered in 70.7%; all six cases of SCC occurred in patients with preoperative suspicion, while LS in 39.3%, and benign inflammatory changes in 12.5% [3]. Pearce et al. also noted that all four cases of SCC were associated with clinical suspicion, while LS was diagnosed in 43% of specimens [4]. These studies, together with our findings, indicate that the likelihood of identifying clinically significant malignancy in the prepuce without suspicious features is exceedingly low, while LS and benign inflammation are the predominant findings.

**Fig. 1:**
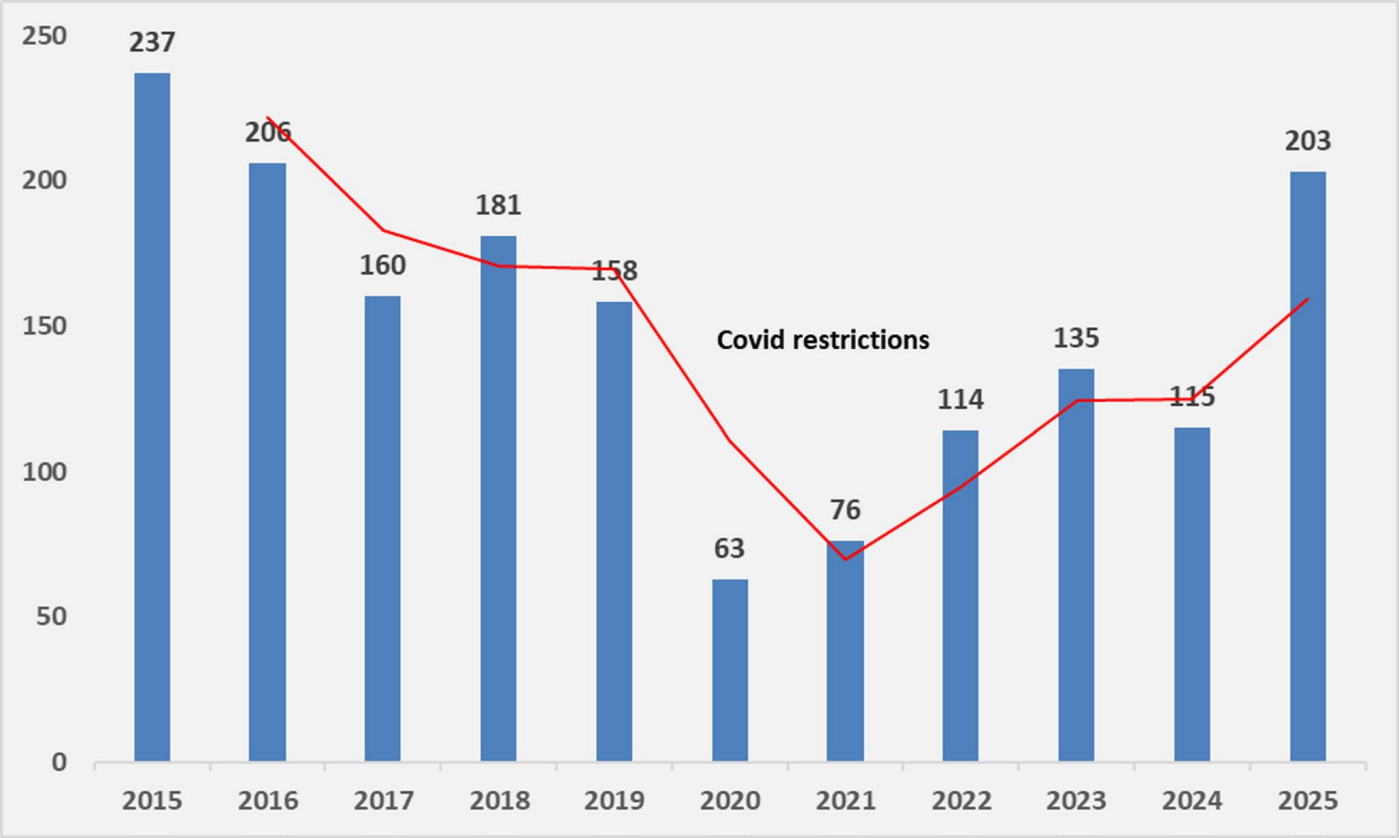
Total number of circumcisions per year (n 1648 without exclusions)

Between 2015 and 2025, a total of 1648 adult circumcisions were performed at our hospital. From 2015 to 2019, an average of 189 procedures were performed annually. However, during the COVID-19 emergency period (2020–2021), this figure declined sharply to an average of 70 procedures per year, as only patients with significant symptoms underwent operations. Following the lifting of COVID restrictions, the numbers increased to 142 per year (2022–2025) but remained lower than pre-pandemic levels due to continued limitations in theatre allocation and a shift in procedural priorities (Fig. 1). Excluding the COVID emergency data, our hospital averaged 168 circumcisions per year, equating to approximately four preputial specimens per week for histopathological assessment. As these specimens are typically low priority, they often accumulate in a backlog. Combined with limited staff resources, this leads to delays, potential errors, and an impact on the timely processing of other urgent diagnostic work.

The financial implications of preputial histopathological examination differ significantly across various healthcare systems worldwide. Costs are influenced by regional policies, institutional tariffs, and whether the service is accessed via public or private healthcare providers. Understanding these variations is essential for both clinicians and patients when considering the resources required for such a procedure. In the United States, the average cost was $311 USD, while Canadian centres reported $150–$200 CAD. The UK’s NHS does not have a fixed tariff, but standard biopsy costs can range from £200 to £400, and private clinics charge between £150 and £230 for histopathology in addition to the procedure cost [1, 13, 15–17].

According to pathology workforce planning guidance, non-complex specimen reporting requires 10–12 min of a consultant pathologist’s time, excluding laboratory staff and transportation times [11, 12]. Our analysis indicated that eliminating routine prepuce histopathology could have saved 304.6 h over 11 years (27.6 h annually). Given that each specimen assessment costs £226, this would provide savings of £344,198 (£31,291 per year). Factoring in laboratory personnel time, administrative time spent by urologists and pathologists, and the increasing demands for urgent cancer diagnostics amid ongoing staffing shortages, these time and cost savings could have a meaningful impact on resource utilisation and departmental efficiency.

Collectively, the available evidence, including our own data, suggests that histopathological examination of the prepuce following routine circumcision in the absence of clinical suspicion is unlikely to detect significant occult malignancy or alter patient management. The incidence of incidental PeIN is very low, and circumcision appears sufficient for its eradication when confined to the foreskin [7, 8]. We advocate for a selective approach, reserving histopathological analysis for cases with abnormal macroscopic findings while emphasising patient education regarding self-examination and prompt reporting of new symptoms. This strategy balances patient safety with judicious use of healthcare resources.

This study is strengthened by its large sample size and long-term follow-up; however, certain limitations merit consideration. The retrospective design may introduce selection bias, and histopathological criteria and reporting practices may vary between institutions, potentially influencing diagnostic rates. Future prospective studies, ideally with standardised protocols and multi-institutional collaboration, are warranted to further define the optimal indications for histopathological examination and to assess the impact of selective strategies on patient outcomes and healthcare efficiency.

## Conclusion

Our findings support a clinical risk-adapted approach to histopathological assessment of the prepuce with substantial cost savings. Routine analysis in the absence of clinically suspicious findings is of limited benefit. Ongoing vigilance through patient education and clinical follow-up remains essential for early detection of potential malignancy.

## Data Availability

No datasets were generated or analysed during the current study.

## Abbreviations

PeIN: Penile intraepithelial neoplasia
LS: Lichen sclerosis
EPR: Electronic patient records
SCC: Squamous cell carcinoma
HPV: Human papillomavirus
